# Parkinson's disease in LGBT+ older adults: The unexplored connection

**DOI:** 10.1016/j.clinsp.2023.100196

**Published:** 2023-05-01

**Authors:** Fulvio A. Scorza, Antonio-Carlos G. de Almeida, Ana C. Fiorini, Carla A. Scorza, Josef Finsterer

**Affiliations:** a Disciplina de Neurociência, Escola Paulista de Medicina, Universidade Federal de São Paulo (EPM/UNIFESP), São Paulo, SP, Brazil; b Centro de Neurociências e Saúde da Mulher “Professor Geraldo Rodrigues de Lima”, Escola Paulista de Medicina/Universidade Federal de São Paulo (EPM/UNIFESP), São Paulo, SP, Brazil; c Laboratório de Neurociência Experimental e Computacional, Departamento de Engenharia de Biossistemas, Universidade Federal de São João del-Rei (UFSJ), São João Del-Rei, MG, Brazil; d Departamento de Fonoaudiologia, Escola Paulista de Medicina, Universidade Federal de São Paulo (EPM/UNIFESP), São Paulo, SP, Brazil; e Programa de Estudos Pós-Graduado em Fonoaudiologia, Pontifícia Universidade Católica de São Paulo (PUC-SP), São Paulo, SP, Brazil; f Neurology & Neurophysiology Center, Vienna, Austria

Research has consistently documented that Lesbian, Gay, Bisexual, and Trans+ (LGBT+) people have poor health outcomes, elevated mortality risks, and higher rates of disease burden than the general population [1]. Given the importance of this data, we read with great interest the article by Crenitte and colleagues addressing priority issues in clinical research describing some of the adverse health and well-being outcomes that older members of LGBT+ communities experience [2]. Considering that these older individuals are at real risk of disabilities, and poor mental and physical health [1,2], it would be interesting to include Parkinson's Disease (PD) in this context.

Most LGBT+ older adults are particularly diagnosed ‒ due to the effects of both cumulative disadvantage and aging - with a chronic condition [3], including PD. The current reality is that the world's population is aging. Proof of that, between 2015‒2030, the number of individuals in the world aged ≥60 years is estimated to grow by 56%, from 901 million to 1.4 billion, and by 2050, the global population is estimated to be more than double the size of 2015, reaching approximately 2.1 billion [4]. In parallel, aging is also the main risk factor for PD, a neurodegenerative disease associated with a significant burden on disability, comorbidities, stigma, costs, and mortality [4]. Importantly, epidemiological studies have shown that PD is accompanied by an increased risk of premature death caused mainly by cardiac abnormalities when compared to the general population [4]. Following this line of reasoning, aging also affects LGBT+ people. Thus, it has been demonstrated that the LGBT+ older adult population is rapidly growing [5]. Current data show that approximately 2.7 million U.S. adults aged 50 and older currently self-identify as LGBT+, including 1.1 million aged ≥65; these figures are estimated to double by the year 2060 [5]. Furthermore, projections made by the U.S. Census estimated that by 2060, those that self-identify as LGBT+ will number nearly 20 million [5]. Based on these data, it is pertinent to speculate that new cases of PD will be diagnosed in LGBT+ people, possibly increasing mortality rates in this population.

On the whole, it is important to note that the LGBT+ older adult population will face several difficulties, such as mental health problems (depression and anxiety), difficulties with body image issues, social isolation (discrimination, social stigma), other chronic physical conditions (heart diseases, hypertension, cancers) and economic disparities. Importantly, our research group is convinced that there is a need to reduce complications of PD in the LGBT+ older adult population. For this purpose, clinical research must be carried out and convergence between health and humanity professionals must be established urgently. Faced with this problem, we have to consider that older LGBT+ people are not a homogeneous group, who have historically faced various obstacles to the fulfilment of their civil rights [6]. These obstacles have been effectively described, including the lack of a legal framework, specifically the lack of laws that protect the rights of this population, failures in the dialogue between the state and civil society, lack of budget forecasts for plans and programs, and lack of political representation in the LGBT+ community [6]. Finally, we are sure that the data obtained in the next studies will have significant implications for public health policy and research, highlighting the importance of medical, social, and behavioral sciences practices in the LGBT+ older adult population.

## Conflicts of interest

The authors declare no conflicts of interest.
